# Marine Diesel Engine Exhaust Emissions Measured in Ship’s Dynamic Operating Conditions

**DOI:** 10.3390/s20226589

**Published:** 2020-11-18

**Authors:** Artur Bogdanowicz, Tomasz Kniaziewicz

**Affiliations:** Faculty of Mechanical and Electrical Engineering, Polish Naval Academy, 81-103 Gdynia, Poland; t.kniaziewicz@amw.gdynia.pl

**Keywords:** ship emissions, artificial neural network, dynamic states, ports, hydrographic survey vessel

## Abstract

The paper presents the results of research on measuring the emissions from marine diesel engines in dynamic states. The problem is as follows: How to measure emissions of the composition of exhaust gases on board a ship, without direct measurement of fuel consumption and an air flow to marine diesel engine, during maneuvering the ship in the port area. The authors proposed a measurement methodology using an exhaust gas analyzer with simultaneous recording of the load indicator, engine speed, inclinometer, and Global Positioning System (GPS) data. Fuel consumption was calculated based on mean indicated pressure (MIP) tests. Recorded data were processed in the LabView systems engineering software. A simple neural network algorithm was used to model the concentrations of ingredients contained in engine exhaust gases during dynamic states. Using the recorded data, it is possible to calculate the emissions of the composition of exhaust gases from the marine diesel engine and calculate the route emissions of the tested vessel.

## 1. Introduction

A typical operation of a ship contains three basic states of operation: Stay at quay, maneuvering, and cruise. During this states, ships perform standard unberthing and mooring maneuvers, in addition they make frequent changes of direction and speed. In ports and offshore areas we can find special units such as tugs and dredgers, in which main propulsions are exposed to variable loads during most of their operation. Load changes of main propulsions affect variable emissions of ingredients contained in engine exhaust gases into the atmosphere. Due to the fact that port areas are close to human agglomerations, maneuvering vessels affect the health of people living there [1,2]. Therefore, the problem of the emissions from marine diesel engine in dynamic states taking place in ports and harbors areas is necessary to be investigated.

In reference [3], the authors comprehensively reviewed the status of the air quality measured in harbor areas. Measured concentrations of the main air pollutants were compiled and intercompared, for different countries worldwide allowing a large spatial representativeness. However, the published studies showed a limited number of available air quality monitoring data in harbor areas, mostly located in Europe.

In Reference [4], a detailed exhaust emission inventory of ships by using Automatic Identification System (AIS) data were developed for Tianjin Port, one of the top 10 world container ports and the largest port in North China. Pollutants were mainly emitted during cruise and hoteling modes, and the highest intensities of emissions located in the vicinity of fairways, berth and anchorage areas in Tianjin Port.

The author of the paper [5] confirmed that the emissions in ports were substantial. In the paper, the ports in Asia and Europe were examined and concluded that most of emissions came from container ships and tankers. It was estimated that approximately 230 million people were directly exposed to the emissions in the top 100 world ports in terms of shipping emissions.

The estimation and analysis of ship emissions in popular tourist ports in Dubrovnik (Croatia) and Kotor (Montenegro) and along the eastern coast of the Adriatic Sea were presented in the paper [6]. There was also a record (2012–2014 years) of cruise ships calling at these ports, which was used to model and estimated exhaust emission factors and their impact on the area of bays and ports.

There is an ongoing debate regarding the measurement of emissions from ships operating at sea. Currently, there are no guidelines or legal requirements defining methods and rules for minimizing the emission of exhaust gases during sea crossing by vessels. In the paper [7], the existing methods for calculating energy consumption and emissions were presented. The authors conceived an attempt to propose the most appropriate method of obtaining the data needed for optimal energy management, which could be applied to any vessel. The considerations mainly concerned the sea crossing.

In Reference [8], the authors presented a method for estimating the emissions of analyzed exhaust gases in ports on the basis of vessel traffic data refer to one year. The research focused mainly on cruise and passenger ships. The analysis concerned only the movement of ships without taking into account auxiliary activities such as maneuvering the vessel or loading at the quay.

In Reference [9], the authors presented the possibility of estimating emissions using the chemical model of transport in the North Sea area. The data for the model were provided by the Dutch research institute MARIN, which is responsible for creating emission reports in the Baltic Sea areas and selected Dutch ports [10,11].

The conducted research on emissions from marine diesel engines is mainly focused on averaging emissions. In the field of aircraft engines, in the paper [12], the model for determining the exhaust emissions of aviation piston engines during the flight of an aircraft was presented. The assessment was carried out in accordance with the guidelines contained in Annex 16 to the Convention on International Civil Aviation Organization (ICAO), while the test covered the operational parameters of the engine corresponding to the approach, landing and operations at the airport.

The civilian ships realizing sea crossing are obliged to comply with provisions on the protection of the marine environment. One of such documents is the International Convention for the Prevention of Pollution from Ships, known as the “MARPOL Convention” with subsequent annexes [13,14,15].

In Reference [16], the authors examined the potential effects of the emerging international maritime emission regulations on the competition between seaports and the potential underlying economic motivations fostering the discussion of introducing Emission Control Areas (ECA). The legal analysis; however, showed that the current enforcement regime of MARPOL Annex VI should be improved in order to rule out the possibility of a low degree of compliance and to protect the competitiveness of complying ships.

In Reference [17], the International Maritime Organization (IMO) is working on development of new short-term measures to implement greenhouse gas (GHG) strategy. Draft new mandatory measures to cut the carbon intensity of existing ships have been agreed by an IMO working group. It is assumed to reduce carbon intensity of international shipping by 40% by 2030, compared to 2008. The amendments were developed by the seventh session of the Intersessional Working Group on Reduction of GHG Emissions from Ships (ISWG-GHG 7), held as a remote meeting 19–23 October 2020.

In the case of warships, they are exempt from compliance with emission standards. However, naval fleets, including the Polish Navy, implement the regulations on their ships as far as possible.

This article is a continuation of authors previous works on the emissions from marine diesel engines. Due to the fact that research on emissions from marine diesel engines is mainly aimed on averaging emissions, the authors decided to measure the emissions more precisely, focusing on their waveforms. The authors decided to use neural networks for modeling emission from marine diesel engines in dynamic states. The neural networks were used in emission modeling [18], but in the field of marine diesel engines there is a gap. The research questions that this article tries to answer are as follows:

- Is it possible to measure and calculate the emissions of the composition of exhaust gases in dynamic operating conditions of marine diesel engine, taking into account the distance travelled?
- Is it possible to use a low-calculation method for modeling the emissions of the composition of exhaust gases in the dynamic operating conditions of the ship’s propulsion system?

## 2. Materials and Methods

### 2.1. Research Object

**Figure 1:**
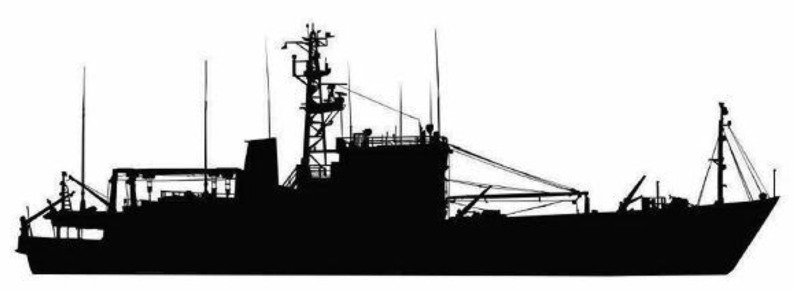
The silhouette of the hydrographic ship [19].

The research was carried out on board the hydrographic ship project 874 (Figure 1) while carrying out survey work at sea. The ship’s data are presented in Table 1.

The main purpose of the ship is to perform hydrographic measurements and research, to put up navigation signs, and to mark the shallows in the sounding areas. The tasks are carried out as part of the navigational and hydrographic security of the Polish Navy and for the maritime economy and navigation safety. 

**Figure 2:**
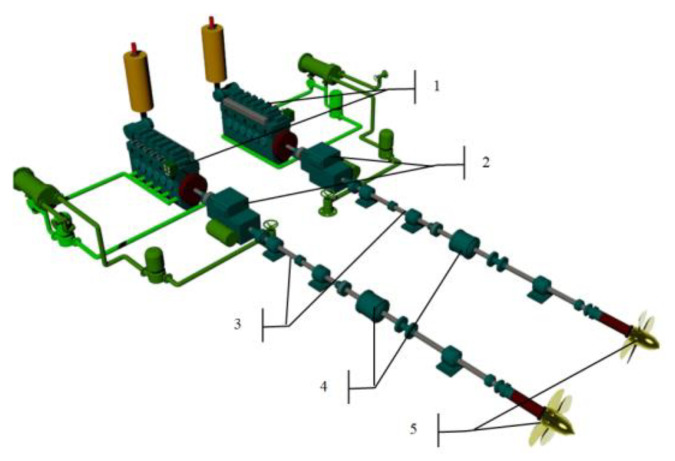
Visualization of the two-shaft main propulsion system with two controllable pitch propellers (CPP): 1—Main diesel engines, 2—reduction gears with auxiliary electric motors, 3—shaft lines, 4—CPP hydraulic boxes, 5—CPP’s.

The hydrographic ship has two engine compartments. In the main engine room, there are two SULZER 6AL25/30 marine diesel engines and one generator set WOLA 39H12 marine diesel engine. In the auxiliary engine room there are the other two generating sets WOLA 39H12 marine diesel engines. The visualization of the main propulsion system is shown in Figure 2.

The research was aimed at measuring the concentration of exhaust gases components from the main propulsion marine diesel engine. The SULZER engine data type 6AL25 / 30 are presented in Table 2.

The measurements were realized using portable exhaust gas analyzer TESTO MARITIME 350 [20]. It can be used to measure the gaseous flue gas concentrations of oxygen (O_2_), carbon monoxide (CO), carbon dioxide (CO_2_), nitrogen oxides (NO_x_), and sulfur dioxide (SO_2_). The technical data of portable analyzer are presented in Table 3.

The GPS receiver type BU-353S4 was used to record the position of the ship. The technical data are presented in Table 4.

The indicator MA2018 was used to measure the indicated pressure. The device was developed at the Polish Naval Academy and allows measurements with a sampling frequency of 20 kHz and with resolution of 12 bits. This device works with a piezoelectric pressure transducer type 7613B KISTLER. Technical data are presented in Table 5. In addition, the device uses a GIG PDS-1 vibration acceleration sensor, which is mounted on the bolts securing the cylinder head cover.

The ADVANTECH USB-4711A unit is equipped with an onboard terminal block, 16-ch analog input, 2-ch analog output, 16-ch digital I/O, and a counter channel capable of outputting a constant frequency square wave [23]. The technical data are presented in Table 6.

The USB-4711A unit was connected with:

- Rotating speed sensor,
- fuel rack sensor, and
- inclinometer.

Rotating speed was measured using reflection sensor Optom OCOE 02581 [24]. The linear potentiometer LPF 150 [25] was used as fuel rack sensor. The Kubler 8.IS40.23321 inclinometer [26] was used to measure the angles of pitch and roll. Measurement for this type of device is carried out biaxially in the ranges of ±60°. The output signal is a voltage ranging from 0 to 5 V for both axes. It is supplied with a voltage ranging from 10 to 30 V. The device housing provides IP68 protection.

**Figure 3:**
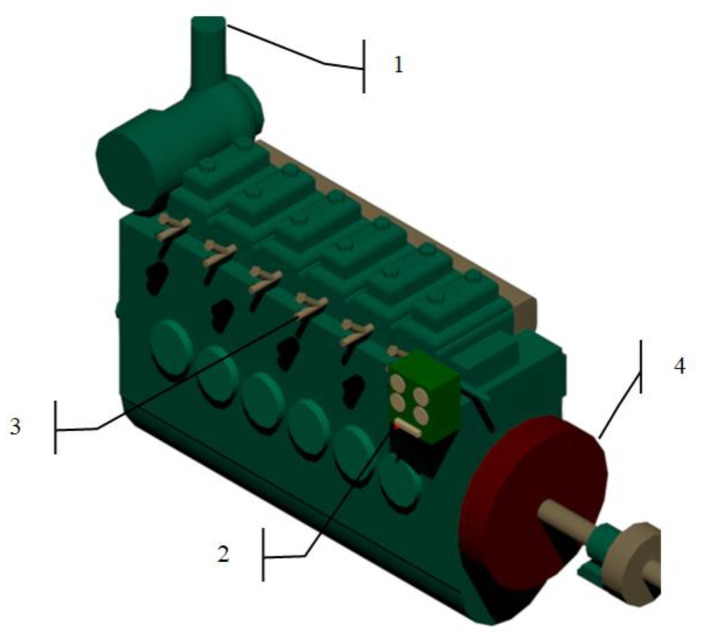
The arrangement of portable measuring transducers on the SULZER type 6AL25/30, 1 –exhaust gas analyzer probe, 2—fuel rack sensors, 3—indicated pressure sensor, 4—rotating speed sensor.

A logging program was written in the Labview [27] system engineering software to record the measurement data. All parameters were recorded with the frequency of 2Hz. Due to TESTO MARITIME 350 analyzer recorded data at a frequency of 1 Hz, there was no need to increase the logging frequency. The portable transducers were mounted on the SULZER marine diesel engine, type 6AL25/30 (Figure 3).

### 2.2. Measurement Site

**Figure 4:**
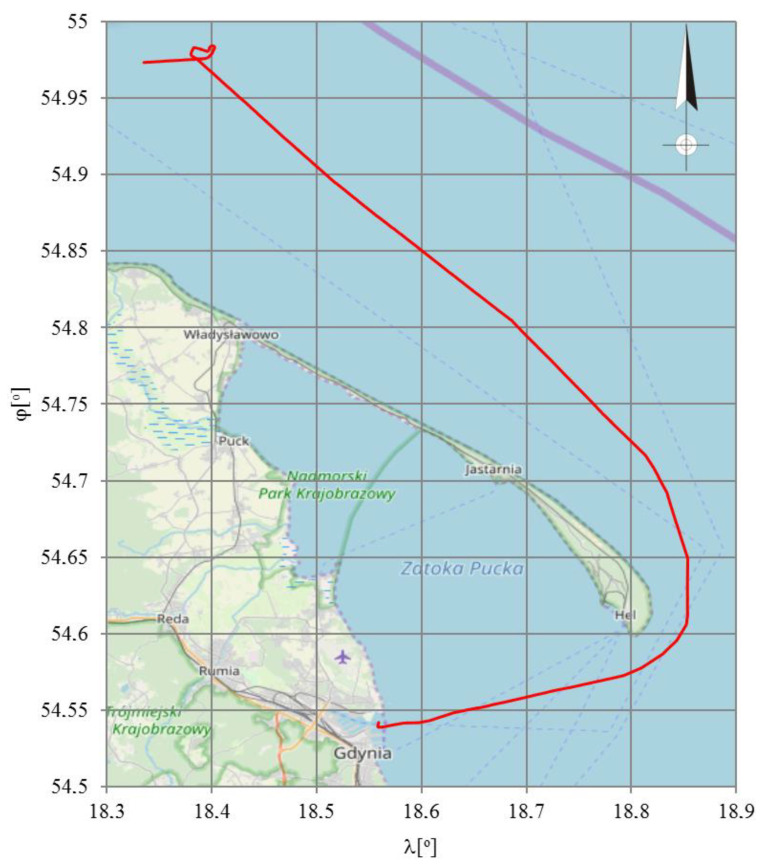
Registered route of the hydrographic vessel.

The measurements were performed in Gulf of Gdansk and the area north of Władysławowo (Figure 4).

The measurement was carried out from the maneuvers in the Navy Port Gdynia to sounding area. The hydrometeorological conditions in the area were as follows:

- Wind direction and strength: NW—4,
- sea state: 3,
- atmospheric pressure: 1017 hPa, and
- air temperature: +5 °C.

Due to the large amount of recorded measurement data, the authors focused only on the ship’s departure from the Navy Port Gdynia.

### 2.3. A Neural Network

The study of the nervous systems is an important factor in the advancement of systems theory and its practical applications. As early as 1943, McCulloch and Pitts developed a model of the nerve cell, the idea of which has survived over the years and is still the basic of most models in use. An important element of this model is the sum of the input signals with an appropriate weight and subjecting the obtained sum to the non-linear activation function [28].

**Figure 5:**
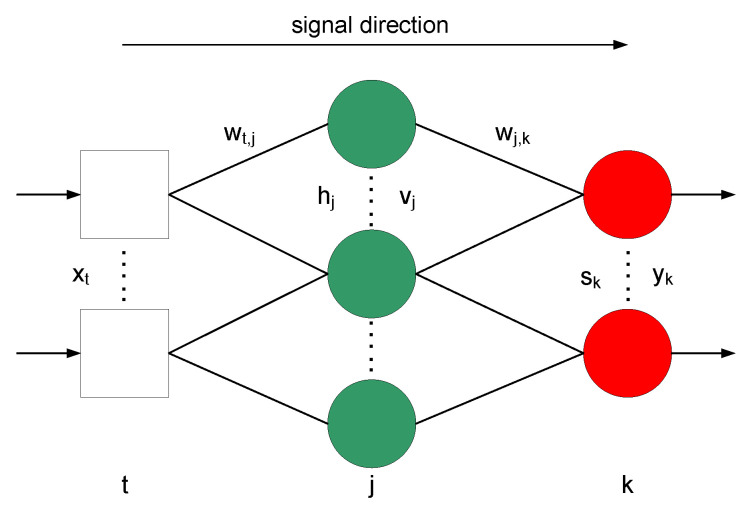
The signal direction in the neural network.

The neural network receives information in the form of numerical variables, which are then sent, taking into account the weighting factors, to the individual neurons. Typically, the activation function is a linear, sigmoidal unipolar or bipolar. In modeling emissions of the composition of exhaust gases from marine diesel engine, it was decided to use the unipolar sigmoid function (Equations (2) and (4)). The products of the weight variables are added up and sent to the next layers. The number of operations depends on the number of neurons in the network. Figure 5 shows a diagram of the operation of a neural network with mathematical relationships used in the approximation.

(1) $$h_{j}=\underset{t}{\sum }w_{t,j}\times x_{t}$$

(2) $$v_{j}=\frac{1}{1+exp\left(-h_{j}\right)}$$

(3) $$s_{k}=\underset{j}{\sum }w_{t,j}\times v_{j}$$

(4) $$y_{k}=\frac{1}{1+exp\left(-s_{k}\right)}$$

Backpropagation was used as the learning algorithm, which changed the weights, taking into account the minimization of errors:

(5) $$\sigma_{k}=y_{k}\times \left(1-y_{k}\right)\times \left(y_{k}^{n}-y_{k}\right)$$

(6) $$\sigma_{j}=v_{j}\times \left(1-v_{j}\right)\times \underset{k}{\sum }\sigma_{k}\times w_{j,k}$$

(7) $$w_{j,k}\left(t+1\right)=w_{t,j}\left(t\right)+\mu \times \sigma_{k}\times v_{j}$$

(8) $$w_{t,j}\left(t+1\right)=w_{j,k}\left(t\right)+\mu \times \sigma_{j}\times w_{j,k}.$$

**Figure 6:**
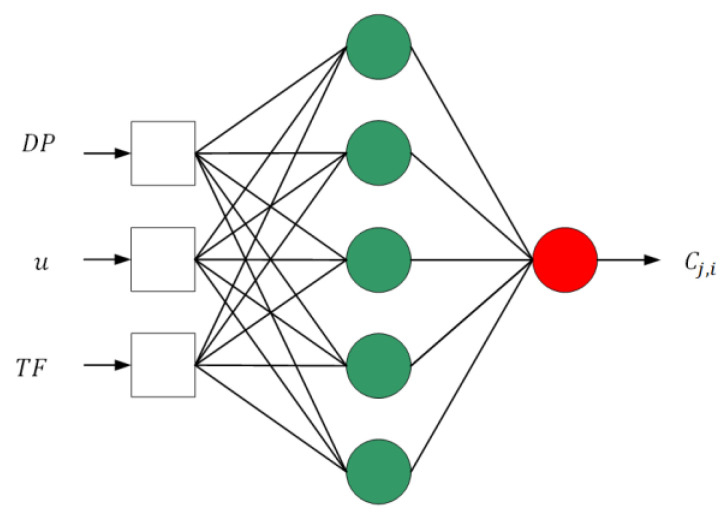
The neural network in configuration 3-5-1. *DP*—the fuel dose $\left[\frac{g}{\mathrm{rot}.}\right]$, *u*—the speed of a ship [knots], *TF*—the exhaust gases temperature [°C], $C_{j,i}$—the concentration of exhaust gases components [ppm, %].

A model of a neural network in the 3-5-1 configuration was built to model the emission concentrations. The choice of this configuration was dictated by the use of the simplest possible network to model emissions in dynamic states. More neurons in the hidden layer could result in the so-called “Overfitting the network” and receiving unreliable results, which would lead to more attempts to learn the neural network. The diagram of the neural network is shown in Figure 6. The network has three inputs, five neurons in the hidden layer, and one output neuron. The sigmoid function was used as the activation function in hidden layer and output neurons.

### 2.4. The Durbin–Watson Test

The Durbin–Watson test was used to check the fit of the model and empirical data [29] which uses the residual differences in values between the data obtained from the model and the empirical. The following relationship was used for the calculations:

(9) $$DW{\;}_{\;}=\;\frac{\sum_{i=1}^{n-1}{\left(R{\;}_{reg\;C_{j}\left(i+1\right)}-R{\;}_{reg\;C_{j}\left(i\right)}\right)}^{2}}{\sum_{i=1}^{n}R_{reg\;C_{j}\left(i\right)}^{2}}$$

where:

n—the number of data items,

$R{\;}_{reg\;C_{j}\left(i\right)}$—the regression residuum value,

$R{\;}_{reg\;C_{j}\left(i+1\right)}$—next step value of the regression residuum.

To determine the value of the Durbin–Watson (DW) test it is necessary to use the DW distribution tables [30]. Low ($d_{l}$) and high ($d_{g}$) limits are specified for the number of predictors and the number of data items in the model. These values determine the range of the residual correlation test. The DW statistic ranges from 0 to 4. The following range of the residual component correlation is assumed:

- if DW = 2, there is no correlation,
- if DW > 2, then
  ◦ if DW > 4 − $d_{l}$, there is a negative correlation,
  ◦ if 4—$d_{g}<DW<$ 4 − $d_{l}$, no conclusion/decision,
  ◦ if DW < 4 − $d_{g}$, no correlation,
- if DW < 2, then
  ◦ if DW < $d_{l}$, there is a positive correlation,
  ◦ if $d_{l}<DW<$ $d_{g}$, no conclusion/decision,
  ◦ if DW < 4 − $d_{g}$, no correlation,

### 2.5. The Emission of the Composition of Exhaust Gases in Dynamic States

Due to the impossibility of installing a fuel flow meter on the ship’s engine, authors decided to calculate the fuel consumption on the basis of MIP measurements. For this purpose, the thermal efficiency was determined for SULZER type A engines. It has been done on the basis of previous ship’s engine tests and preliminary tests carried out on a laboratory engine SULZER, type 6AL20/24. The indicated power was calculated using the indicated pressure:

(10) $$N_{icyl}=\frac{V_{s}\cdot n\cdot p_{i}\cdot z}{60}$$

where:

z—number of ignitions (for 4-s engines z = 0.5),

$V_{s}$—displacement volume$\;\left[m^{3}\right]$,

$p_{i}$—mean indicated pressure [Pa], and

n—rotation speed $\left[\min^{-1}\right]$.

The fuel mass flow rate consumed by a diesel engine was calculated:

(11) $${\dot{m}}_{fuel}\;=\;\frac{\sum_{i=1}^{k}N_{icyl}}{W_{d}\;\cdot \eta_{i}}\;\left[\frac{\mathrm{kg}}{s}\right]$$

where:

$\eta_{i}$—thermal efficiency,

$W_{d}$—the calorific value of a fuel (for the NATO F–75 fuel is $W_{d}$ = 42,700,000 $\frac{J}{\mathrm{kg}}$), and

k—number of cylinders.

The fuel dose for one cylinder during one work cycle was calculated:

(12) $$DP=\;\frac{{\dot{m}}_{fuel}\cdot 60,000}{n\;\cdot k}\left[\frac{\mathrm{kg}}{\mathrm{rot}}\right]$$

where:

n—rotation speed $\left[\min^{-1}\right]$.

The fuel dose was linearly dependent on the load indicator:

(13) DP=f(WO)

The air mass flow rate and exhaust mass flow rate were calculated from the actual air demand, taking into account the excess air factor λ:

(14) $$L_{R}=\lambda \;\times \left[11.84\times c+34.214\times h\right]\;\left[\frac{{\mathrm{kg}}_{\mathrm{air}}}{{\mathrm{kg}}_{\mathrm{fuel}}}\right]$$

(15) $$\lambda =\frac{20.95\;}{20.95-\;C_{O_{2}}}$$

(16) $${\dot{m}}_{air}={\dot{m}}_{fuel}\;\times L_{R}\;\left[\frac{\mathrm{kg}}{s}\right]$$

(17) $${\dot{m}}_{ex}={\dot{m}}_{fuel}+{\dot{m}}_{air}\;\left[\frac{\mathrm{kg}}{s}\right]$$

where:

$C_{O_{2}}$—oxygen concentration in exhaust gases [%].

The composition of the NATO F–75 fuel used in the Polish Navy is c = 0.87 i h = 0.13.

The intensity of mass emissions of individual components were calculated on the basis of the equation:

(18) $$E_{i,j}=u_{j}\times C_{i,j}\times {\dot{m}}_{ex}$$

where:

${\dot{m}}_{ex}\;—$exhaust mass flow rate $\left[\frac{\mathrm{kg}}{s}\right]$,

$C_{i,j}—$the concentration of exhaust gas components [ppm, %],

$u_{j}$—factor characteristic for a given compound j:

$u_{CO}$ = 0.000966, $u_{CO_{2}}$ = 15.19, $u_{NO_{x}}$ = 0.001587

The emissions of components were calculated by integrating the intensity of mass emissions over time:

(19) $$m_{i,j}=\int_{t_{pp}}^{t_{kp}}E_{i,j}dt\;\left[\mathrm{kg}\right]$$

where:

$t_{pp}$—the beginning of the dynamic state [s],

$t_{kp}$—the end of dynamic state [s].

Based on the measurement data from the GPS system, the distance traveled was calculated from the relationship (20) using the law of cosines:

(20) $$d=\mathrm{acos}(\sin\left(\varphi_{1}\right)\times \sin\left(\varphi_{2}\right)+\cos\left(\varphi_{1}\right)\times \cos\left(\varphi_{2}\right)\times \cos\left(\lambda_{2}-\lambda_{1}\right)\cdot R$$

where:

$\varphi_{1}$—the latitude of the first point,

$\varphi_{2}$—the latitude of the second point,

$\lambda_{1}$—the longitude of the first point,

$\lambda_{2}$—the longitude of the second point, and

R—radius of the Earth.

The route emission is used to assess the ecological properties of ships in terms of the emission of exhaust gases components. It is used as a reference quantity for the distance traveled by the ship, in emission models and emission inventories. The ship’s route emissions were calculated from the following relationship:

(21) $$b_{s}=\frac{m_{i,j}}{d}\;\left[\frac{\mathrm{kg}}{\mathrm{NM}}\right]$$

The presented calculation algorithm was implemented in the LabView development environment. By this way, it was possible to analyze any interval of the recorded experiment. The next part of the paper presents the results of the analysis of maneuvers carried out by the hydrographic ship during departure the Navy Port Gdynia.

## 3. Results

The departure of the hydrographic ship was divided into four stages:

Stage 1—unberthing and hauling off maneuver,
stage 2—moving off maneuver,
stage 3—change of direction maneuver, and
stage 4—acceleration maneuver.

**Figure 7:**
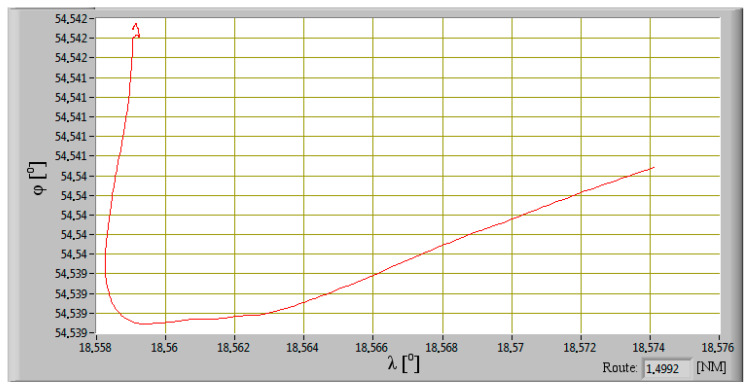
Recorded way during the ship departure from the port.

The ship way in port is shown in Figure 7. In the first stage, the main propulsion engines were coupled to a line of shafts driving propellers. The ship’s propulsion operated at a constant rotation speed of 600 min^−1^. The CPP’s were set to 0. At this stage, the engines of the generator sets were mainly loaded, which provided electricity for the working bow thruster. After unberthing and hauling off maneuver, the ship was changed course to heads of breakwater and moved off. By the CPP’s changing, the main propulsion engines were loaded and the ship reached an ahead speed of 4 knots which kept until change of direction maneuver. The ship turned to port, causing the vessel roll to starboard. At the time of turning, the ahead speed of the ship was slightly reduced. After establishing a course towards the entrance, the ship began accelerating to the ahead speed of 11 knots.

**Figure 8:**
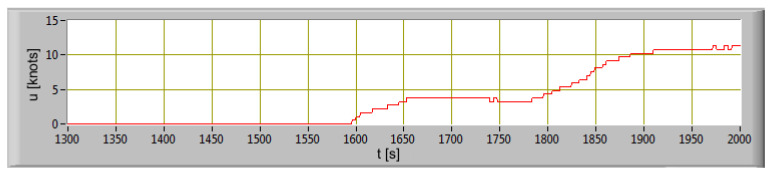
The ship’s speed change during the departure from the port as a function of time.

Table 7 shows values of the distance traveled which were calculated on the basis of data obtained from the GPS system. The ship’s speed changes during the departure from the port is presented in Figure 8.

**Figure 9:**
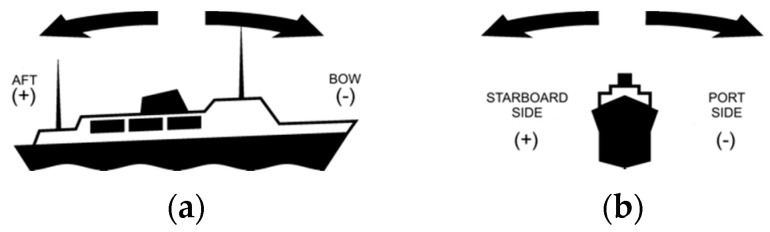
Marks of pitch (**a**) and roll (**b**) changes.

The pitch and roll of the ships were recorded using an inclinometer, which was mounted on the main propulsion engine (port side). Admit mark of direction of pitch and roll are shown in Figure 9.

**Figure 10:**
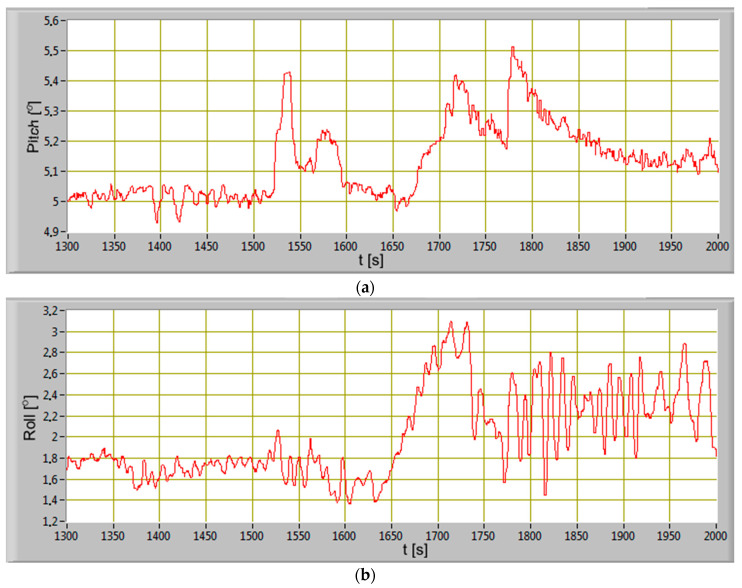
The ship’s pitch (**a**) and roll (**b**) change during the departure from the port as a function of time.

The changes of the ship’s pitch and roll during departure from the port are shown in Figure 10. The ship alongside the berth had starboard side list and small trim by the stern.

**Figure 11:**
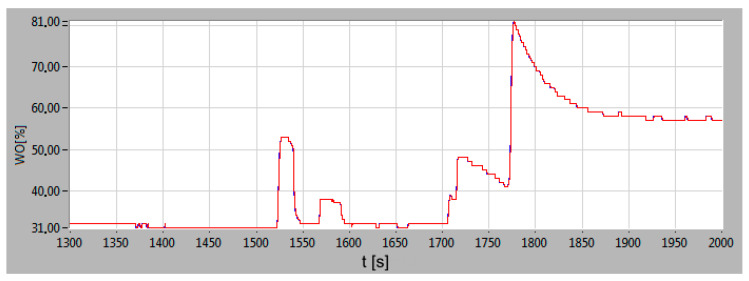
The main engine (port side) load indicator change during the departure from the port as a function of time.

The change of main engine load indicator is shown in Figure 11. In stage 1, the engine load was constant because the ship was maneuvering only with the bow thruster. The first change in the main engine load (increase) occurred while accelerating the vessel to the speed of 4 knots. After reaching the set speed, the main engine load stabilized at the load indicator of 32%. The change of direction forced a temporary, slight decrease in the value of the ship’s speed and an increase in the load to 48%. This was caused by the helm angle to the port side, causing an increase in the hydrodynamic resistance of the left side propeller and the hull. At the same time, the ship heeled to starboard. In order to maintain a constant main engine rotational speed, the governor forced an increase the fuel dose injected into the cylinders and was recorded on the waveform. The last increase in the load to the value of 81% was caused by the acceleration of the ship to a cruising speed of 11 knots. At the same time, a temporary change in trim towards the stern (immersion of the propeller) and a slight stabilization were recorded.

**Figure 12:**
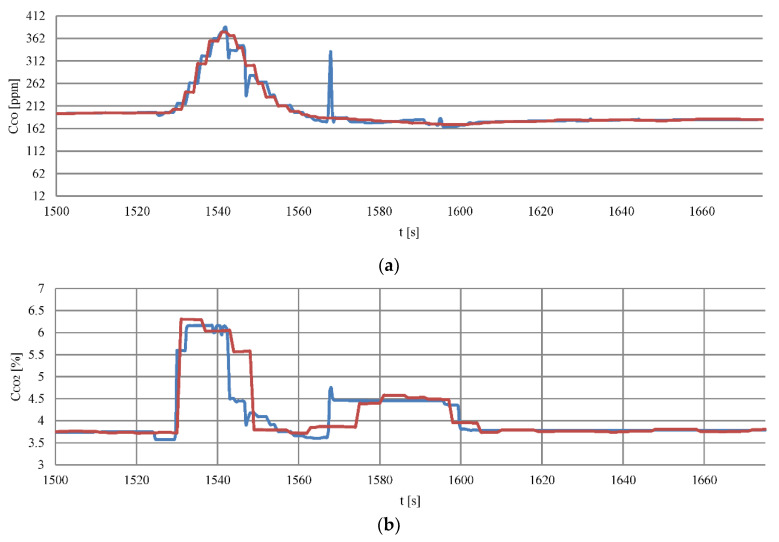

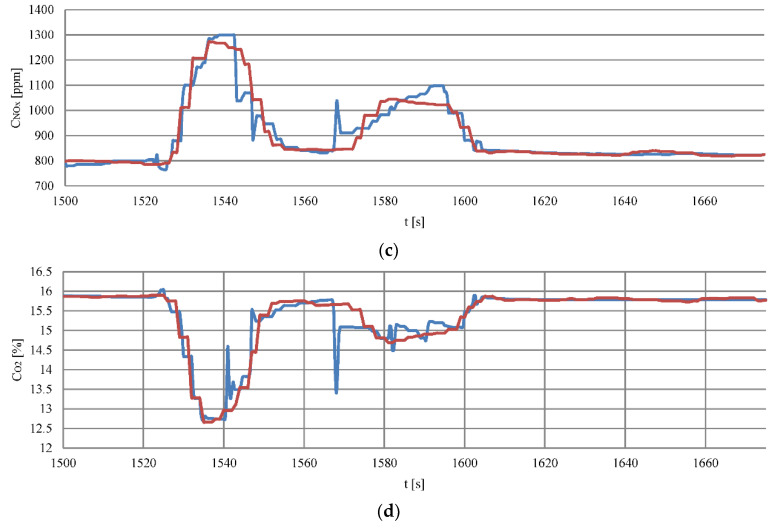
The courses of changes concentrations: (**a**) Carbon monoxide, (**b**) carbon dioxide, (**c**) nitrogen oxides, (**d**) oxygen during moving off maneuver as a function of time, red line—recorded data, blue line—data from the model.

Changing the CPP’s (connected with the engine load) caused a change concentrations of analyzed substances in exhaust gases. The courses of changes in individual recorded and calculated on the basis of the neural net’s concentration model are presented in Figure 12.

**Figure 13:**
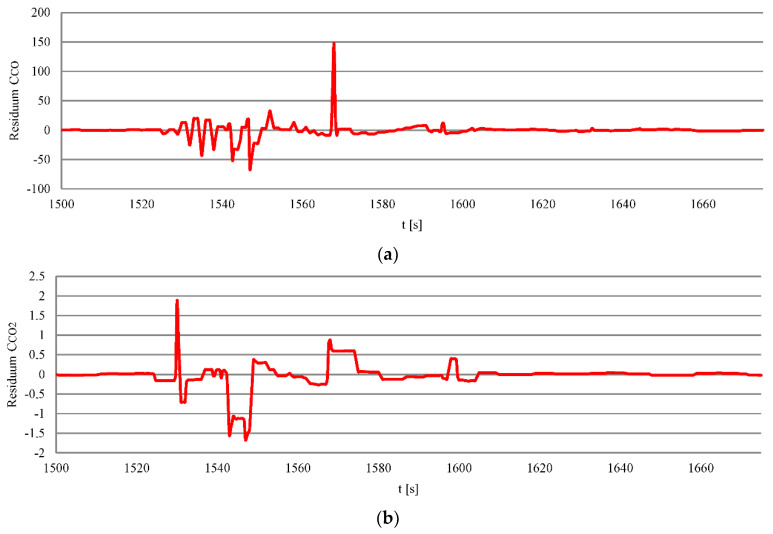

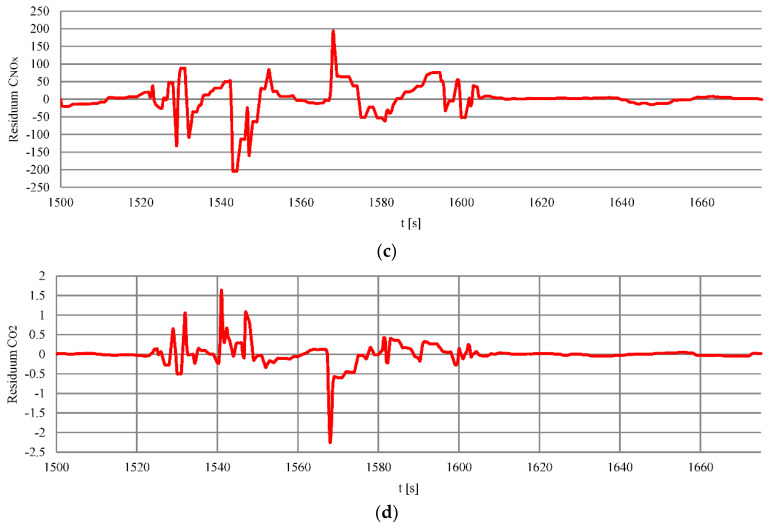
The courses of changes regression residuum: (**a**) Carbon monoxide, (**b**) carbon dioxide, (**c**) nitrogen oxides, (**d**) oxygen during moving off maneuver as a function of time.

The recorded and calculated data regression differences were used for comparison (Figure 13). The Regression residuum were subjected to the Durbin–Watson test, described in the previous section.

The modelling of the concentrations of the composition of exhaust gases were performed only for stages 2 to 4. In stage 1 the ship was maneuvering with the bow thruster and only the generator sets were loaded. This fact was considered irrelevant in terms of modelling dynamic states.

The regression residuum of the composition of exhaust gases was subjected to the DW test. The calculated values show that the model and empirical data show a strong positive correlation at the studied stages. Table 8 shows the values of the regression residuum of the model and empirical data during the departure maneuver from the port.

**Figure 14:**
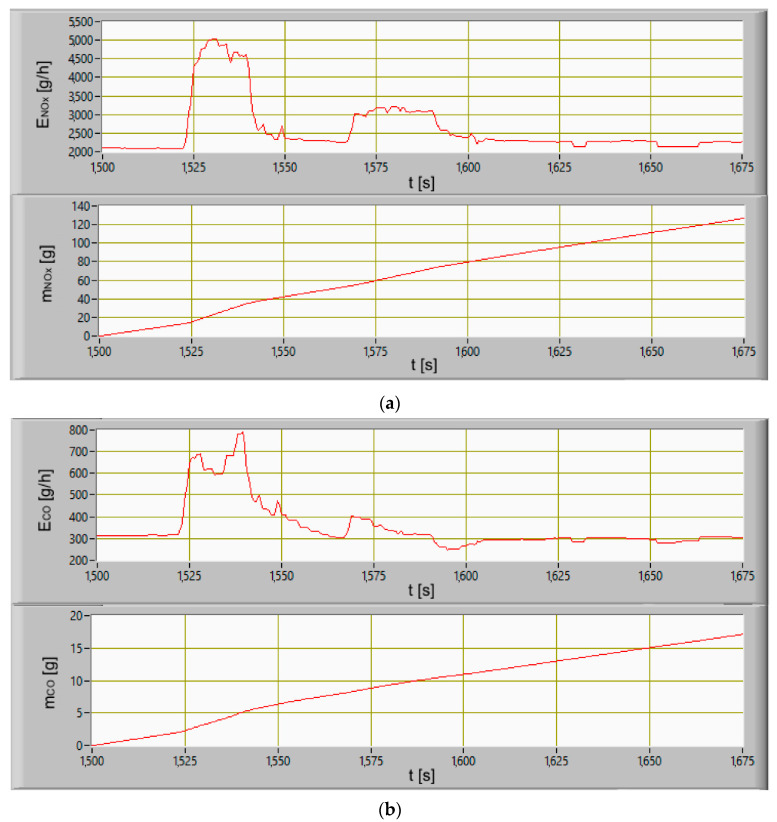

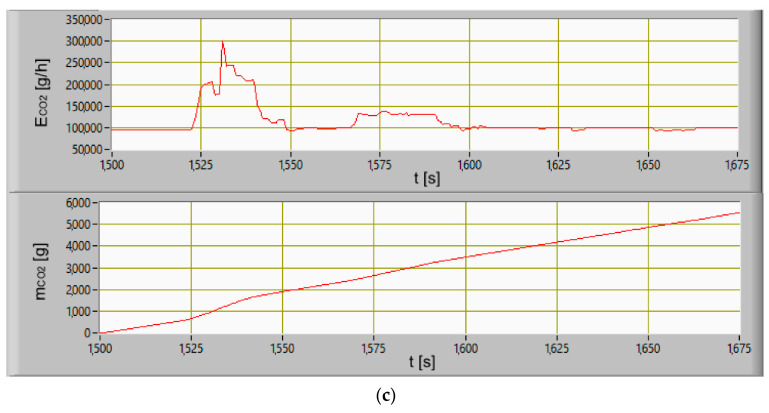
The courses of changes emissions intensity and emissions of exhaust gases components: (**a**) Nitrogen oxides, (**b**) carbon monoxide, (**c**) carbon dioxide during moving off maneuver as a function of time.

On the basis of the concentrations’ courses of carbon monoxide, carbon dioxide, and nitrogen oxides, the intensity of mass emissions of the exhaust gases components were calculated. Numerical integration of the intensity of mass emissions courses made it possible to obtain a curve showing the waveform in emissions during the maneuvers of the hydrographic ship in the port. In Figure 14 the waveforms of the emissions intensity and the emissions of the exhaust gases components during the moving off maneuver of the ship in the port (stage 2) are presented. Analyzing the waveforms, it can be concluded that the fastest reaction to diesel engine load changes can be seen in the change of the nitrogen oxides concentration. In order to maintain the set engine speed during load changes, the governor increased fuel doses of all cylinders, which resulted in an increase in the fuel-air mixture combustion temperature. The sudden engine load change caused a delay in the operation of the turbocharger as a result of incomplete combustion. There was an increase in the carbon monoxide emission intensity in the exhaust gases. The slowest reaction to a load change is seen in carbon dioxide concentration change. By adjusting the ship’s speed, the crew forced a smaller change in the engine load and propulsion system. The intensity of mass emissions gradually stabilized after the ship reached the cruising speed set. The stabilization of the engine load and the intensity of mass emissions resulted from the cooperation between the hull and the propulsion system of the ship. The stabilization of the ahead speed of the ship was resulted of equal resistances of the hull components and the thrust force of the propellers. The ship, performing the maneuvers in the port, was sheltered from the hydrometeorological conditions in Gulf of Gdansk. In stage 4, the ship was leaving the port and began to be affected by the waves coming from the waters of the gulf. It was recorded (t = 1750 s to t = 2000 s) in the waveforms of the vessel’s pitch and roll angles (Figure 10), the engine load indicator (Figure 11).

**Figure 15:**
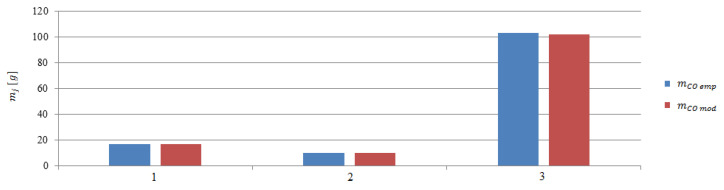
Carbon monoxide emissions from model and empirical data during the maneuvers: 1—Moving off, 2—change of direction, 3—acceleration.

**Figure 16:**
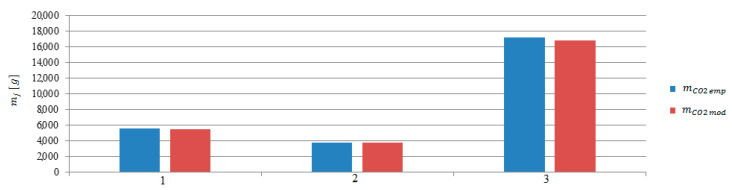
Carbon dioxide emissions from model and empirical data during the maneuvers: 1—Moving off, 2—change of direction, 3—acceleration.

**Figure 17:**
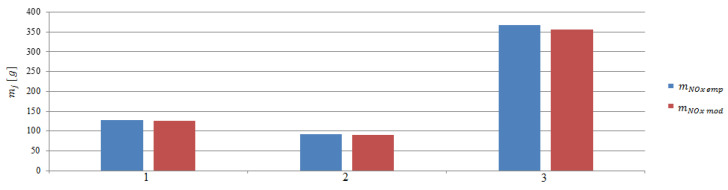
Nitrogen oxides emissions from model and empirical tests during the maneuvers: 1—Moving off, 2—change of direction, 3—acceleration.

The next step was to calculate the emissions in stages 2 to 4. The calculations were made on the basis of model and empirical data (Figure 15, Figure 16 and Figure 17). The performed calculations show that the highest values of the exhaust gases components were emitted during the last stage of departure from the port. The lowest values were recorded during changing the direction maneuver. The moving off maneuver to 4 knots (stage 2) slightly increased the values compared to the change of direction maneuver.

**Figure 18:**
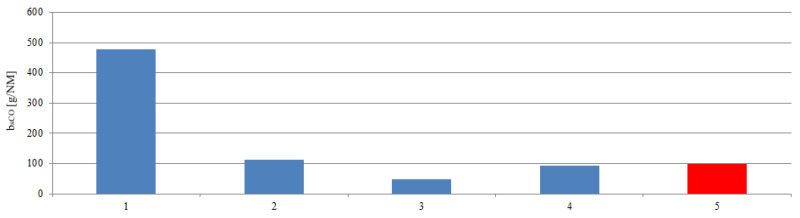
The ship’s route carbon monoxide emissions during the stages: 1—Unberthing and hauling off maneuver, 2—moving off maneuver, 3—change of direction maneuver, 4—acceleration maneuver, 5—mean value of the ship’s route emission.

**Figure 19:**
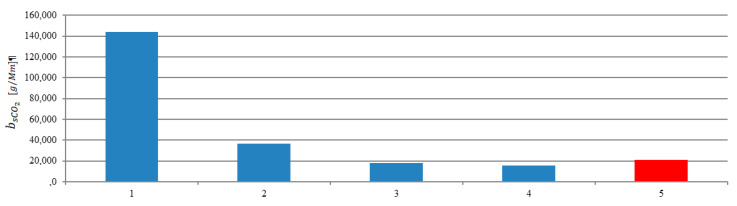
The ship’s route carbon dioxide emissions during the stages: 1—Unberthing and hauling off maneuver, 2—moving off maneuver, 3—change of direction maneuver, 4—acceleration maneuver, 5—mean value of the ship’s route emission.

**Figure 20:**
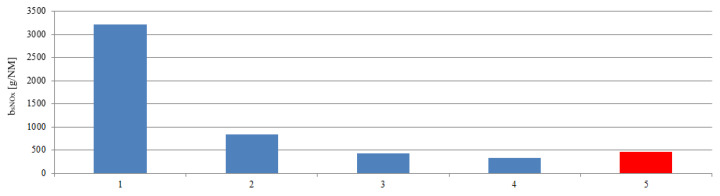
The ship’s route nitrogen oxides emissions during the stages: 1—Unberthing and hauling off maneuver, 2—moving off maneuver, 3—change of direction maneuver, 4—acceleration maneuver, 5—mean value of the ship’s route emission.

The determination of the emissions and the ship’s route made it possible to calculate road emissions. In these calculations, the stage 1 was taken into account. The emission of the main engine operated at a constant load was designated. The ship’s route emissions are presented in Figure 18, Figure 19 and Figure 20. The red bar shows the calculated mean value of the ship’s route emission for the entire range connecting all maneuvers performed by the ship in the port. In stage 1 and 2 emission reached values greater than the average, while the stage 3 and 4 emission were lower than the average value.

## 4. Conclusions

The results of the experiment confirmed that it is possible to calculate the emission of exhaust gases components from the main propulsion engine in the dynamic states. If it is not possible to measure fuel consumption directly, indirect methods can be used to obtain these values from MIP measurements. The use of simple neural networks make it possible to model the concentrations of the exhaust gases components in dynamic operating states. They are the input data for the calculation of the emissions of exhaust gases components from the marine diesel engine. The presented experiment took place during a routine task of the hydrographic survey vessel. Due to large amount of registered data, only the ship’s departure maneuvers from the Navy Port Gdynia is presented. The results of the experiment led to general conclusions:

1. Carrying out tests of multi-engine propulsion system, the measurement set should be extended with additional sensors and an exhaust gases analyzer allowing for the parallel measurement of all engines.
2. Approximation with the use of neural networks gives exact results of the fit, that was confirmed by the analysis of regression residuum using the Durbin–Watson test.
3. The learned neural networks will be used to estimate the emissions of exhaust gases in a model of ship’s propulsion. This solution will enable to build enhanced model that will estimate emission in port and coastal areas traffic.
4. The ship’s route emissions for a vessel maneuvering at place or at low speed are higher than for vessels realizing passage at cruising speeds.
5. Active slowing down (not described in the paper) involving the operation of the propulsion system “backwards” while the unit is moving forward, also increases the value of the ship’s route emissions than in the case of passive slowing down of the ship.
6. In the experiment, the position was recorded using the GPS system. The assumption was to obtain the sampling time every 1 s. In future works, the AIS system collecting information about the movement of vessels in the port will be used. This will allow the emissions of ships maneuvering in port areas to be calculated. However, the system registration time is longer than the direct GPS measurement.

The proposed methodology of dynamic emissions tests in port areas can be performed without interfering with the ship’s propulsion system (in particular the fuel system of the marine diesel engine). The presented devices and methods of analysis will be further developed. It is planned to determine the influence of hydrometeorological conditions on the change the emissions of exhaust gases components from marine diesel engine. The research should be extended to ship boilers and generator sets, which also operate when the unit’s propulsion system is stopped or is in idle gear while the ship is maneuvering or moored to the quay.

## Figures and Tables

**Table 1:** Project 874 ship data [19].

Specification	
Standard displacement	1145 t
Full displacement	1214 t
Length overall	61.6 m
Breadth	10.8 m
Draught	3.3 m
Speed	13.7 w
Main engines	2 × SULZER type 6AL25/30
Generators	3 × WOLA39H12

**Table 2:** Marine diesel engine SULZER type 6AL25/30 [19].

Specification	
Piston arrangement	Inline
Cylinder diameter	250 mm
Piston stroke	300 mm
Displacement volume	1 cyl.—14.726 dm^3^
Nominal power	706.08 kW
Starter	pressure compressed air—3 MPa
Number of cylinders	6
Number of valves per cylinder	4

**Table 3:** Parameters and measuring ranges of the TESTO 350 analyzer [20].

Parameter	Measuring Range	Tolerance
Temperature range	from −40 to + 1000 °C	max. ± 5 °C
Oxygen range	from 0 to 25%	According to MARPOL, Annex VI or NO_x_ Technical Code
Carbon monoxide range	from 0 to 3000 ppm	
Nitrogen monoxide range	from 0 to 3000 ppm	
Nitrogen dioxide range	from 0 to 500 ppm	
Sulphur dioxide range	from 0 to 3000 ppm	
Carbon dioxide range	from 0 to 40%	
Absolute pressure range	from 600 to 1150 hPa	±5 hPa w 22 °C ±10 hPa w −5 do +45 °C

**Table 4:** Antenna GPS type BU-353S4 [21].

Specification	
Chipset	SiRF STAR IV GSD4e
Frequency	L1; 1575.42 MHz
C/A code	1.023 MHz chip rate
Channels	48
Sensitivity	−163 dBm
Accuracy position	<2.5 m 2D RMS SBAS Enable
Velocity	0.1 m/s
Time	1 µs synchronized to GPS time

**Table 5:** Pressure transducer type 7613B KISTLER [22].

Specification	
Pressure range	0–25 MPa
Maximum indicated pressure	30 MPa
Sensitivity	2 mV/MPa
Resonant frequency	60 kHz
Temperature range	223–623 K
Temperature drift 473, …, 500 K	3.5%

**Table 6:** ADVANTECH USB-4711A unit [23].

Specification	
Channels	16 analog input, 2 analog output
Resolution	12 bits
Max. sampling rate	150 kS/s max
FIFO size	1024 samples
Sampling mode	Software, onboard programmable pacer, and external
Bipolar Absolute Accuracy (% of FSR)	±10 ± 5 ± 2.5 ± 1.25 ± 0.625 0.1 0.1 0.2 0.2 0.4

**Table 7:** Ship’s distance in the port.

The Stage	Distance [NM]
No. 1	0.039
No. 2	0.15
No. 3	0.21
No. 4	1.1
Sum:	1.499

**Table 8:** The Durbin–Watson test values for the concentration of exhaust gases components regression residuum.

The Stage	The Concentration	The Durbin–Watson Test
No. 2	$C_{CO}$	0.694121033
	$C_{CO_{2}}$	0.215164508
	$C_{NO_{x}}$	0.203601472
	$C_{O_{2}}$	0.390373856
No. 3	$C_{CO}$	0.467848069
	$C_{CO_{2}}$	0.857018339
	$C_{NO_{x}}$	0.463965328
	$C_{O_{2}}$	0.626851209
No. 4	$C_{CO}$	0.471728494
	$C_{CO_{2}}$	0.868353646
	$C_{NO_{x}}$	0.149970084
	$C_{O_{2}}$	0.778670739
